# Therapeutic Potential of Edaravone for Neuroprotection Following Global Cerebral Hypoxia

**DOI:** 10.3390/ijms26189019

**Published:** 2025-09-16

**Authors:** Johanna Franziska Busse, Jonas Frai, Luca Ines Hamacher, Veronika Matschke, Carsten Theiss, Thomas Weber, Jennifer Herzog-Niescery, Sarah Stahlke

**Affiliations:** 1 Institute of Anatomy, Department of Cytology, Ruhr-University Bochum, 44801 Bochum, Germanyveronika.matschke@rub.de (V.M.);; 2 International Graduate School of Neuroscience (IGSN), Ruhr-University Bochum, 44801 Bochum, Germany; 3 Department of Anesthesiology and Intensive Care Medicine, St. Josef-Hospital Bochum, 44791 Bochum, Germany

**Keywords:** hypoxia, edaravone, neuroprotection, oxidative stress, sex differences in brain injury

## Abstract

Global cerebral hypoxia triggers (mal-)adaptive responses that can lead to neuronal damage. This study evaluated edaravone’s neuroprotective effects in a rat hypoxia model, focusing on sex differences, treatment durations, and behavioral outcomes. Male and female rats underwent global cerebral hypoxia induced by rocuronium, with post-hypoxia edaravone treatment. Motor coordination and activity were assessed through exploratory behavior tests. Histological analyses evaluated neuronal integrity and apoptosis, while microglial activity and gene expression were analyzed via immunofluorescence and qPCR. Edaravone showed transient neuroprotective effects on motor behavior and early immune responses, particularly in the cerebellum and hippocampus. No gross morphological damage was observed, though functional impairments occurred despite preserved cytoarchitecture. Microglial activity was initially suppressed in treated and later activated in untreated hypoxic brains, suggesting modulating immune responses. Gene expression analysis revealed region-specific, time-dependent, and sex-specific changes, including early upregulation of CCR7, S100B, and NSE in treated animals. Males were more susceptible to hypoxic damage, while females showed higher baseline resistance and better functional recovery. Seven-day edaravone treatment increased apoptotic markers in male cerebellum, indicating sex-specific differences in cell death mechanisms. These findings highlight the potential for personalized therapy and underscore the importance of considering sex differences in both research and clinical practice.

## 1. Introduction

The brain is particularly vulnerable to oxygen deficiency, which is reflected in significant neuronal damage even after short periods of cerebral hypoxia. Pathophysiologically, this is explained by its high metabolic rate combined with low oxygen reserves, which both ensure a continuous oxygen supply. Thus, oxygen deficiency stops oxidative phosphorylation, forcing the cells to switch to the more inefficient anaerobic metabolism, which produces far less adenosine triphosphate (ATP). The ATP lack results in intracellular imbalances such as increased calcium levels, which triggers enzymatic and structural damage, and leads to excessive neurotransmitter release (e.g., glutamate), which is known to be neurotoxic in high concentrations [1,2]. In addition, reactive oxygen species (ROS) and lactate accumulate, which both exacerbate cellular stress [3]. At this point, rising oxygen levels cause further cellular damage by activating multiple destructive pathways, including reactive oxygen and nitrogen species-guided damage of cell membranes, mitochondria, and deoxyribonucleic acid (DNA) [4]. The radicals also disrupt the blood–brain barrier, leading to inflammation and immune activation, including the release of second messengers such as S100β [5,6]. Neuron-specific enolase (NSE) is a glycolytic enzyme that is mainly expressed in neurons. It is an established clinical marker of neuronal damage and is often found to be elevated following hypoxic injury [7]. Additionally, CCR7, a chemokine receptor found on immune cells, is upregulated during neuroinflammatory processes, playing a pivotal role in leukocyte migration and central nervous system immune surveillance [8]. Elevated calcium ion levels further amplify ROS production and activate destructive enzymes [4]. Neurons are particularly sensitive to oxygen decline, while oligodendrocytes and microglia are more vulnerable to combined hypoxia and hypoglycemia compared to astrocytes [9,10]. Hypoxia triggers a canonical transcriptional response across brain cell types, but also cell-specific processes [9].

From a clinical perspective, cerebral hypoxia can occur either systemically, affecting the whole body due to insufficient oxygen levels in the blood (hypoxaemia, e.g., caused by perinatal asphyxia, near-drowning, carbon monoxide poisoning, or cardiac and/or respiratory arrest), or locally, caused by vascular occlusion, despite normal oxygen levels in the blood. The treatment depends on the underlying cause; however, it is limited and the outcome often poor, especially for global cerebral hypoxia, which is not accessible to mechanical procedures such as thrombectomy [11].

A promising drug for patients suffering from global cerebral hypoxia might be edaravone, a substance with antioxidant and anti-inflammatory effects. Its beneficial effects in patients after acute ischemic stroke have been summarized in a systematic review recently, stating that edaravone can reduce the ROS concentration in the penumbra after an ischemic insult. In addition, it inhibits neutrophil and microglial activation and reduces the increase in oxidative markers in the early phase of reperfusion, which finally results in reduced neuronal cell death and prevention of brain edema [12]. Although not investigated in patients after global cerebral hypoxia, its antioxidant properties have further been demonstrated in patients with Amyotrophic Lateral Sclerosis (ALS) [13]. Some studies suggest that edaravone treatment may slow ALS progression; correspondingly, data from ALS mouse models reported a reduction in motor loss and nitration of tyrosine residues in the cerebrospinal fluid [14,15]. In Parkinson’s disease models, edaravone protected dopaminergic neurons against 6-OHDA-induced toxicity both in vitro and in vivo, with effects mediated through anti-apoptotic, anti-oxidative, and anti-inflammatory pathways [16]. Following traumatic brain injury, edaravone increased neural stem cell proliferation around damaged areas and enhanced neurosphere formation, suggesting enhanced neuroregeneration potential [17]. Additionally, edaravone provided mitochondrial protection in human neuroblastoma cells against zinc oxide nanoparticle-induced toxicity through heme oxygenase-1-dependent mechanisms, preserving ATP levels and reducing oxidative damage [18].

Given the limited therapeutic options for global cerebral hypoxia and the established neuroprotective properties of edaravone in other conditions, we investigated the potentially beneficial effects of edaravone treatment after global cerebral hypoxia in an in vivo rat model. This model is based on the neuromuscular-blocking drug rocuronium, which leads to severe respiratory insufficiency with consecutive hypoxemia and global cerebral hypoxia while the cerebral blood flow still remains [19]. We hypothesized that edaravone treatment would mitigate hypoxic injury in a rat model, primarily by reducing oxidative stress and neuroinflammation. Specifically, we hypothesized that edaravone would enhance motor coordination and activity, preserve neuronal integrity, and modulate microglial activation in key brain regions, including the hippocampus, cerebellum, and primary motor cortex. Secondary endpoints included the investigation of sex-specific responses to hypoxia and edaravone treatment, as well as the impact of treatment duration on outcomes. In order to adhere to the principles of the 3Rs (replacement, reduction, and refinement), the study design minimized animal use and optimized experimental conditions to ensure ethical and scientific rigor. The observed differences in susceptibility to hypoxic damage and response to edaravone between males and females highlight the potential for personalized therapeutic strategies, underscoring the clinical relevance of the findings.

## 2. Results

### 2.1. Motor Coordination and Activity Assessment After 7-Say Edavarone Treatment

**Figure 1:**
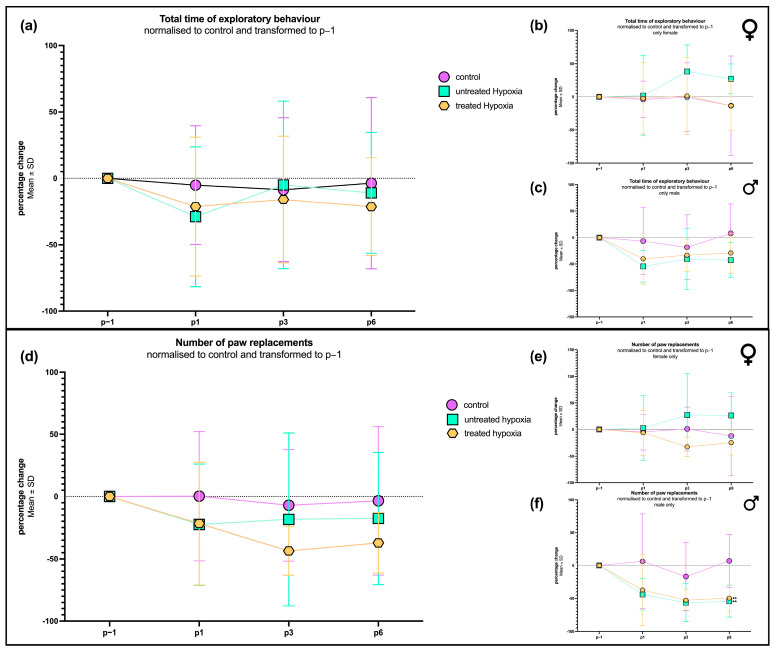
Exploratory behavior in rats following chemically induced hypoxia and treatment with edaravone. (**a**) Percentage change (mean ± SD) in total time spent in an upright position in the cylinder test, measured at baseline (p − 1) and at 1, 3, and 6 days post-intervention (P1, P3, P6). (**b**) Subgroup analysis for female rats. (**c**) Subgroup analysis for male rats. (**d**) Percentage change (mean ± SD) in number of paw replacements in the cylinder test, measured at baseline (p − 1) and at 1, 3, and 6 days post-intervention (P1, P3, P6). Values were normalized to the control group (anesthetized only, no hypoxia) and transformed relative to baseline. (**e**) Subgroup analysis for female rats. (**f**) Subgroup analysis for male rats. *n* = 11–12 for general analysis and *n* = 5–6 for sex-specific subgroups.

To evaluate motor deficits resulting from hypoxia, we examined exploratory behavior and the frequency of paw replacements. No significant difference in exploratory behavior between the hypoxia-exposed animals (with and without edaravone treatment) and the control group could be observed over the entire course of time. On postoperative day 1 (P1), animals that were treated with edaravone after hypoxia exhibited a slightly longer exploration time (−21.28 ± 52.28%) compared to the untreated hypoxia group (52.64 ± 52.28%; Figure 1a). By P3, hypoxia group’s exploratory behavior returned to levels comparable to controls (−8.584 ± 54.12%), while the edaravone-treated group showed a slight reduction (−15.97 ± 47.68%). On P6, the exploratory behavior in the edaravone group remained slightly below both the hypoxia-only (−10.93 ± 45.49%) and control groups (−3.674 ± 64.49%).

In terms of contact frequency with the cylindrical wall (“paw replacements”), both the hypoxia and edaravone-treated groups exhibited a distinct reduction compared to the control group on P1. By P3 and P6, the untreated hypoxia group showed recovery to near-control levels, while the edaravone group displayed a further decline, reaching the lowest wall interactions, with percentage change of −37.15 ± 24.22% on P6.

To investigate potential sex-related differences, the dataset was subdivided into male and female rats. In males (Figure 1c), exploration times mirrored the patterns observed in the combined group. Both hypoxia-exposed subgroups exhibited a pronounced reduction in exploration time on P1, with the untreated group showing the lowest value on P1 (−54.58 ± 29.57%). On P1, the edaravone-treated group demonstrated a moderate increase (−40.31 ± 47.64%) in exploration compared to the untreated hypoxia group (−54.58 ± 29.57%), though both remained below control (−6.544 ± 63.59%) levels across all time points.

In contrast, female rats (Figure 1b) displayed a different behavioral profile. On P1, exploration times were comparable across all groups, with no significant differences observed. However, by P3 and P6, females in the untreated hypoxia group exhibited increased (37.92 ± 40.22%; 27.07 ± 22.55%) exploration time compared to both the edaravone-treated (1.388 ± 57.91%; −13,40 ± 37.60%) and control groups (−0.4601 ± 51.75%; −13.40 ± 74.97%). The latter two groups remained consistent with each other across all measured time points.

Additionally, the number of paw replacements was analyzed across biological sex groups. In males (Figure 1f), the progress closely mirrored those observed in exploration time. Both hypoxia-exposed groups exhibited a reduction (untreated: −44.09 ± 23.97%; treated: −37.30 ± 54.29%) in the total number of paw replacements compared to controls (6.305 ± 72.31%) on P1. The edaravone-treated males showed a modest increase (−49,57 ± 19.94%) relative to untreated hypoxia animals (−54.49 ± 24.36%), although this was still below control levels (7.021 ± 40.41%) on P6.

The lowest number of touches was observed for all male groups on postoperative day 3 (P3), with the following means: control (−16.85 ± 51.75%), untreated hypoxia (−56.68 ± 28.40%), and treated hypoxia (−52.74 ± 16.68%). Although the behavior of the hypoxia groups on P6 was similar to that on P3, significantly fewer replacements were measured in the treated (*p* = 0.0147) and untreated (*p* = 0.0085) hypoxia groups compared to the control group.

In females (Figure 1e), the number of paw replacements followed a similar pattern to their exploratory behavior described above. On P1, all three groups exhibited comparable replacement counts. By P3, the hypoxia group showed an increase in tactile activity, reaching a percentage change of 27.59 ± 78.35%; meanwhile, the edaravone-treated group (−12.82 ± 51.12%) remained similar to the control group (1.129 ± 41.19%). This pattern persisted through P6, where the edaravone group reached its lowest value by a percentage change of (−24.73 ± 22.86%), falling below both the hypoxia (26.60 ± 42.44%) and control groups (−12.21 ± 74.77%).

### 2.2. Structural Changes in Key Brain Regions Following Hypoxic Injury

**Figure 2:**
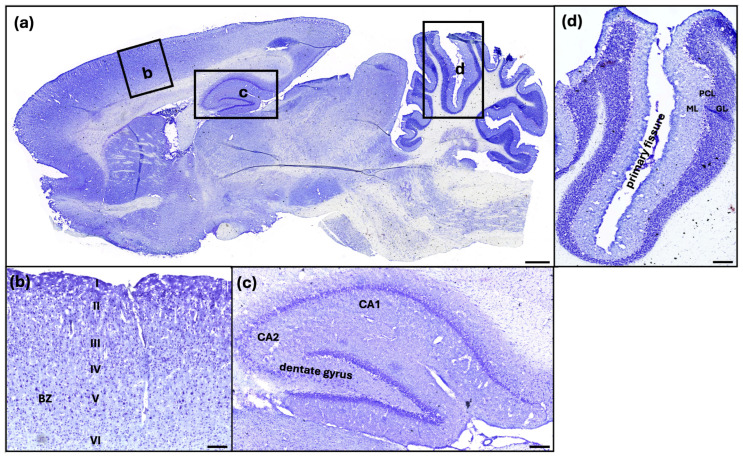
Sagittal section of a control rat brain in cresyl violet staining (**a**). The investigated regions of the primary cortex (**b**), the hippocampus (**c**), and the cerebellum (**d**) are highlighted. I-VI: layer of the isocortex; BZ: betz cell; GL: granule layer; PCL: Purkinje cell layer; ML: molecular layer; scale bar in (**a**) 1 mm, (**b**) 200 µm, (**c**) 200 µm, (**d**) 250 µm.

**Figure A1:**
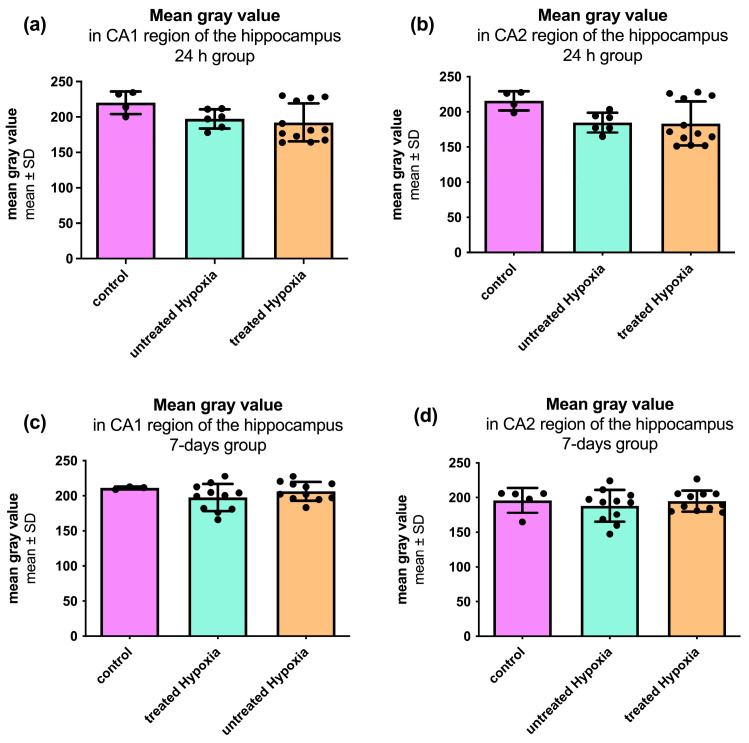
Quantitative analysis of cresyl violet staining intensity in CA1 and CA2 subfields of the hippocampus. Bar graphs show the mean gray value as a measure of cresyl violet staining intensity in (**a**) CA1 and (**b**) CA2 regions for the 24 h group, and in (**c**) CA1 and (**d**) CA2 regions for the 7-day group. Each graph compares control, untreated hypoxia, and treated hypoxia animals. Lower gray values indicate reduced cellular density or integrity. Data are presented as mean ± SD.

**Figure A2:**
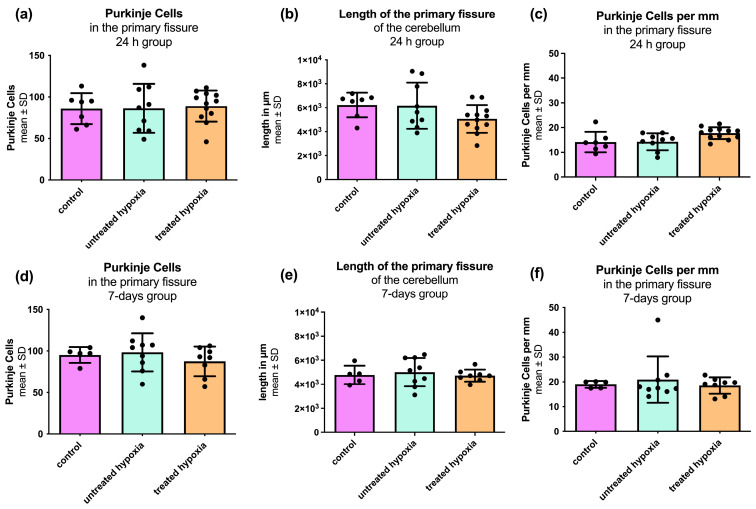
Morphological analysis of the cerebellum with focus on Purkinje cells using cresyl violet staining. Bar graphs show quantification of Purkinje cell morphology in the primary fissure of the cerebellum for 24 h (**a**–**c**) and 7-day (**d**–**f**) groups. Total number of Purkinje cells (**a**,**d**), length of the primary fissure (**b**,**e**), and Purkinje cell density expressed as the number of Purkinje cells per millimeter of fissure length (**c**,**f**). Each panel compares control, untreated hypoxia, and treated hypoxia animals. Data are presented as mean ± SD.

**Figure A3:**
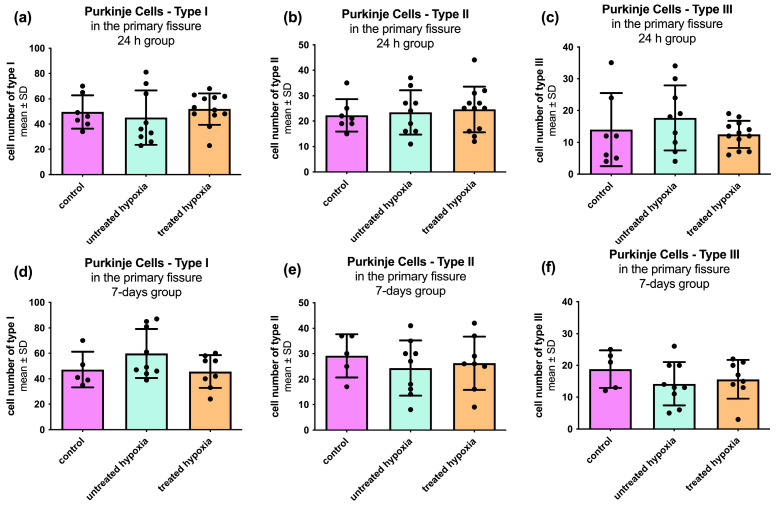
Qualitative classification of Purkinje cells in the cerebellum based on morphological integrity. Bar graphs show the absolute number of Purkinje cells classified into three morphological types based on cresyl violet staining in the primary fissure of the cerebellum. (**a**–**c**) Short-term group; (**d**–**f**) long-term group. (**a**,**d**) Type 1: intact morphology with clearly visible nucleus and cytoplasm; (**b**,**e**) Type 2: moderate morphological alterations (e.g., shrinkage, pale staining); (**c**,**f**) Type 3: severely damaged or degenerated cells. Each graph compares control, untreated hypoxia, and treated hypoxia animals. Data are presented as mean ± SD.

**Figure A4:**
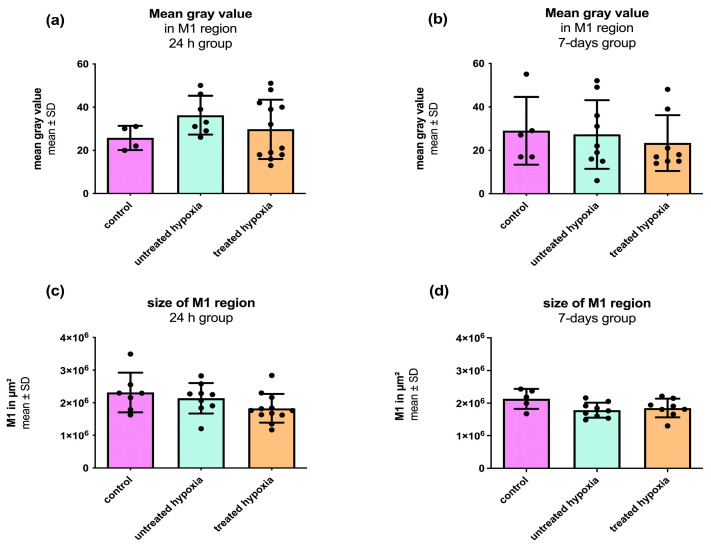
Quantitative assessment of cortical density and size in the M1 region using cresyl violet staining. Bar graphs show the (**a**,**b**) mean gray value as a measure of cortical cell density and (**c**,**d**) total area (in µm^2^) of the M1 region in cresyl violet-stained brain sections. (**a**,**c**) represent 24 h animals; (**b**,**d**) represent 7-day animals. Each graph compares control, untreated hypoxia, and treated hypoxia groups. Data are presented as mean ± SD.

To assess region-specific structural changes following hypoxic injury, we focused on three key brain regions known for their vulnerability to oxygen deprivation and functional clinical relevance: the hippocampus, the cerebellum, and the primary motor cortex (M1). These areas were identified and analyzed based on their distinct cytoarchitecture, as visualized by cresyl violet staining (see Figure 2). No significant differences were observed in the structural integrity of these regions between the control, untreated hypoxia, and edaravone-treated hypoxia groups in either the 24-h or 7-day interventions. Detailed data are provided in Figure A1, Figure A2, Figure A3 and Figure A4.

### 2.3. Microglial Activity Changes After Hypoxia and Can Be Restored by Edaravone Treatment

**Figure 3:**
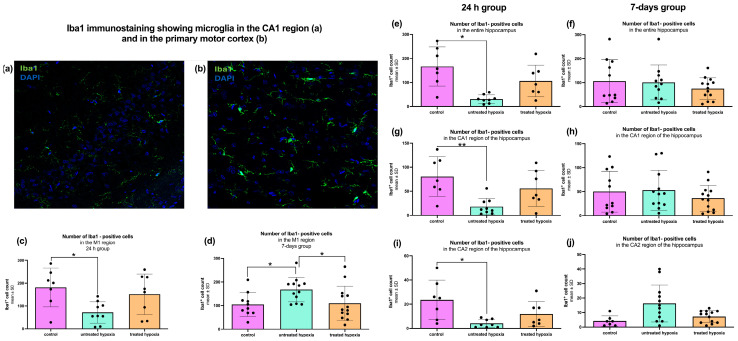
Quantification of *Iba1*-positive microglial cells in the hippocampus and the primary motor cortex of 24 h and 7-day groups. *Iba1* immunostaining (green) showing microglia in the CA1 region of the hippocampus (**a**) and in the primary motor cortex (M1) (**b**). All images represent 24 h untreated hypoxia animals. Nuclei are counterstained with DAPI (blue). Scale bar: 25 µm. Bar graphs on the left-hand side show the number of Iba1-positive cells in the M1 region for the (**c**) 24 h group and (**d**) 7-day group. On the right-hand side the bar graphs show (**e**) the entire hippocampus, (**g**) the CA1 region, and (**i**) the CA2 region for the 24 h treated group, and in (**f**) the entire hippocampus, (**h**) the CA1 region, and (**j**) the CA2 region for the 7-day treated group. Each graph compares three experimental conditions: control (pink), untreated hypoxia (mint), and treated hypoxia (orange). Data are presented as mean ± standard deviation (SD) and significance levels are set at *p* < 0.05 indicated by *, *p* < 0.001 indicated by **. In 24 h treated groups *n* = 7–9 and in 7-day treated groups *n* = 11–13.

The 24 h hippocampal samples were analyzed regarding microglial activation, via *Iba1* immunofluorescence staining (Figure 3). Quantitative analysis of the total hippocampal *Iba1* signal revealed a significant decrease in the untreated hypoxia group compared to the control group (30.88 ± 17.40; 166.6 ± 81.76; *p* < 0.0014; Figure 3e). No other significant differences were observed between the remaining subgroups (control: 166.6 ± 81.76; untreated: 30.88 ± 17.40; treated: 106.3 ± 66.05).

In the 7-day group, no significant differences in total hippocampal *Iba1* signal were detected across any of the experimental subgroups (control: 106.2 ± 90.08; untreated: 100.8 ± 73.10; treated: 74.62 ± 45.04; Figure 3f). To determine the location of inflammatory activity more precisely, the hippocampal area was subdivided into its two anatomical and histological regions CA1 and CA2.

Quantitative analysis of the CA1 region (control: 80.57 ± 41.12; untreated: 18.22 ± 17.12; treated: 56.00 ± 37.27) revealed a significant decrease in the *Iba1* signal in the 24 h untreated hypoxia group compared to the controls (*p* < 0.0059; Figure 3g). A similar decrease in microglial activation was observed in the CA2 region (*p* < 0.0027; control: 23.57 ± 16.34; untreated: 4.13 ± 3.56; treated: 11.86 ± 10.40; Figure 3i). The 7-day group revealed a different pattern in the analysis of the CA1 region, with no significant differences (control: 50.00 ± 42.63; untreated: 52.92 ± 41.49; treated: 36.23 ± 26.91; Figure 3h). Additionally, a moderate increase in the CA2 region of the untreated hypoxia group compared to the controls could be observed (*p* = 0.084; control: 4.14 ± 3.53; untreated: 16.25 ± 12.76; treated: 7.23 ± 4.11; Figure 3j).

The second region analyzed in this study was the primary motor cortex (M1; Figure 3c,d). Quantitative analysis of 24 h tissue samples revealed a significant decrease in *Iba1*-positive cells in the untreated hypoxia group compared to the control group (control: 181.1 ± 85.21; untreated: 71.67 ± 46.91; treated: 159.3 ± 92.43; *p* < 0.0265; Figure 3c). Additionally, a moderate decrease in *Iba1*-positive cells was observed in the untreated hypoxia group compared to the edaravone-treated group, though this was not statistically significant (71.67 ± 46.91 vs. 159.3 ± 92.43; *p* = 0.09).

In contrast, analysis of the 7-day group showed a significant increase in *Iba1*-positive cells (control: 106.9 ± 50.11; untreated: 167.8 ± 51.70; treated: 109.3 ± 72.41) in the untreated hypoxia group compared to the control group (*p* < 0.0375; Figure 3d). Furthermore, the untreated hypoxia group also exhibited significantly more *Iba1*-positive cells than the edaravone-treated group (*p* < 0.0314).

### 2.4. Analysis of General Oligodendrocyte Function and Axonal Insulation via MBP

To assess general oligodendrocyte function and axonal insulation, we performed immunofluorescence staining for Myelin Basic Protein (MBP), which is a key marker of myelination. The analyzed regions were the hippocampus (CA1 and CA2) and the cerebellum. To estimate myelination, the mean gray value was measured in each region.

**Figure 4:**
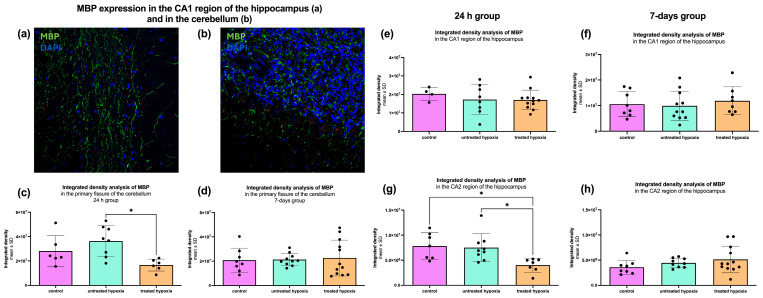
Integrated density of MBP immunoreactivity in hippocampal subregions and the primary fissure of the cerebellum. MBP staining (green) illustrates myelination in the CA1 region of the hippocampus (**a**) and in the cerebellum (**b**), reflecting white matter integrity. Bar graphs on the left-hand side show the integrated density of MBP staining in the primary fissure for (**c**) the 24 h group (*n* = 7–9) and (**d**) the 7-day group (*n* = 8–12). On the right hand-side, bar graphs show the integrated density of MBP staining in the CA1 region: (**e**) 24 h (*n* = 4–12) and (**f**) 7-day groups (*n* = 10–12), and in the CA2 region: (**g**) 24 h (*n* = 7–9) and (**h**) 7-day groups (*n* = 8–13). Each graph compares three experimental conditions: control (pink), untreated hypoxia (mint), and treated hypoxia (orange). Data are presented as mean ± SD and significance levels are set at *p* < 0.05 indicated by *.

In the CA1 region of the hippocampus, no significant differences in MBP signal intensity were observed in either the 24 h treated or the 7-day treated post-intervention groups (24 h treated control: 2.03 × 10^7^ ± 3.51 × 10^6^; untreated: 1.72 × 10^7^ ± 8.04 × 10^6^; treated: 1.70 × 10^7^ ± 5.31 × 10^6^/7-day treated: 1.06 × 10^7^ ± 4.96 × 10^6^; untreated: 9.90 × 10^6^ ± 5.71 × 10^6^; treated: 1.19 × 10^7^ ± 5.44 × 10^6^; Figure 4e,f). However, in the CA2 region for the 24 h treated group, a significant reduction in myelination represented in mean gray value reduction was detected in both the edaravone-treated hypoxia group (4.003 × 10^6^) and the untreated hypoxia group (7.528 × 10^6^) compared to control group (7.841 × 10^6^), with *p*-values of 0.0099 and 0.0126 (24 h control: 7.84 × 10^6^ ± 2.64 × 10^6^; untreated: 7.53 × 10^6^ ± 2.77 × 10^6^; treated: 4.00 × 10^6^ ± 1.38 × 10^6^/7-day control: 3.60 × 10^6^ ± 1.38 × 10^6^; untreated: 4.46 × 10^6^ ± 912,816; treated: 5.14 × 10^6^ ± 2.65 × 10^6^; Figure 4g,h).

In the cerebellum, the 24 h group (control: 2.83 × 10^7^ ± 1.26 × 10^7^; untreated: 3.63 × 10^7^ ± 1.25 × 10^7^; treated: 1.67 × 10^7^ ± 4.88 × 10^6^) showed a significant increase in MBP signal in the untreated hypoxia group compared to the edaravone-treated group (3.63 × 10^7^ vs. 1.67 × 10^7^; *p* = 0.0114; Figure 4c). In the 7-day group, no significant differences were identified (control: 2.08 × 10^7^ ± 1.00 × 10^7^; untreated: 2.13 × 10^7^ ± 5.10 × 10^6^; treated: 2.26 × 10^7^ ± 1.47 × 10^7^; Figure 4d).

### 2.5. Assessment of Neuronal Integrity and Apoptosis in the Brain

**Figure 5:**
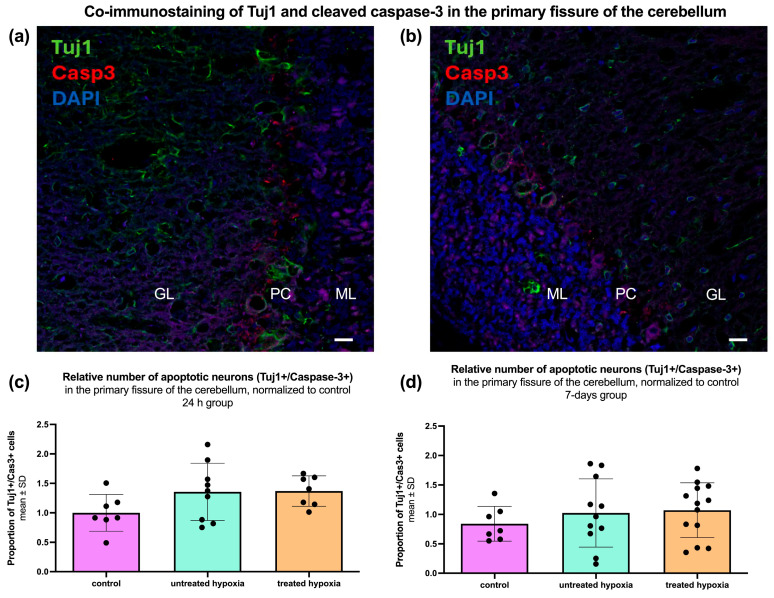
Relative number of apoptotic neurons in the primary fissure of the cerebellum. Co-immunostaining of Tuj1 (green, neuronal marker) and cleaved Caspase-3 (red, apoptosis marker) in the cerebellum, indicating neuronal integrity and apoptotic activity, respectively (**a**,**b**). Bar graphs show the number of Casp3/Tuj1-positive cells in the primary fissure of the cerebellum for the (**c**) 24 h group (*n* = 7–9) and (**d**) 7-day group (*n* = 8–13). Each graph compares three experimental conditions: control (pink), untreated hypoxia (mint), and treated hypoxia (orange). Data are presented as mean ± SD. GL = granular cell layer; PC = Purkinje cell layer; ML = molecular layer.

Immunofluorescence staining for Tuj1 and activated Caspase-3 (Casp3) was performed to assess neuronal integrity and apoptosis in the cerebellum, with the percentage of Casp3+/Tuj1+ double-labeled cells relative to the total number of Tuj1+ cells calculated to evaluate potential treatment effects (Figure 5). Quantitative analysis of the 24 h group (control: 1.00 ± 0.31; untreated: 1.36 ± 0.49; treated: 1.37 ± 0.26) revealed no significant change in the proportion of Casp3+/Tuj1+ double-labeled cells (Figure 5c).

Quantitative analysis of the cerebellum in the 7-day group (control: 0.84 ± 0.29; untreated: 1.03 ± 0.58; treated: 1.07 ± 0.47) revealed no significant differences in neuronal density or apoptosis (Figure 5d). However, when the groups are divided by gender, a significant (*p* = 0.0172) increase in the proportion of Casp3+/Tuj1+ double-labeled cells in male hypoxia animals (2.29 ± 0.72) compared to the corresponding control (0.999 ± 0.439) was found, which is reversible (*p* = 0.358) with edaravone treatment (1.71 ± 0.77).

### 2.6. Hypoxia Markers in the Hippocampus, Cerebellum, and Cortex: 24 h vs. 7-Day

In addition to classical and immunohistochemical staining, mRNA was analyzed using RT-PCR for various marker expression in brain areas (e.g., hippocampus and cerebellum) and blood due to its clinical and diagnostic relevance.

#### 2.6.1. Hippocampus

**Figure 6:**
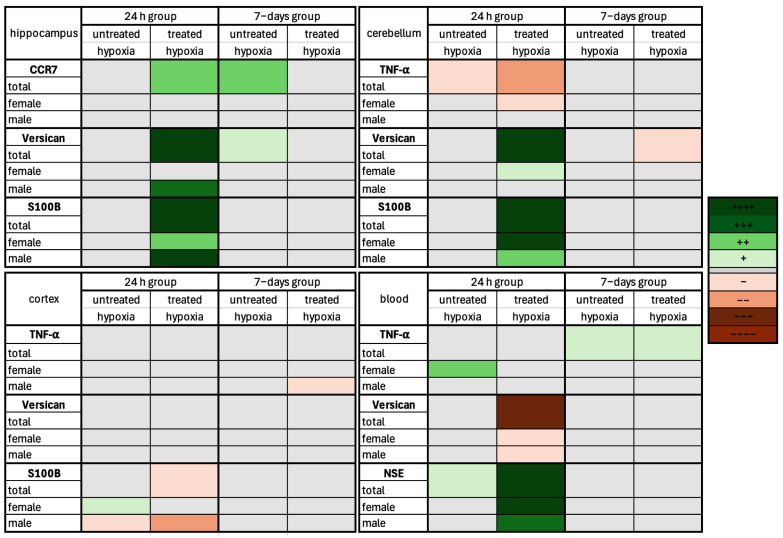
Heatmap visualization shows changes in gene expression for an inflammatory and ECM remodeling marker, as well as a clinical marker, in three brain regions and peripheral blood following hypoxia. This exemplary heatmap shows the relative levels of mRNA expression in hippocampal, cerebellar, and cortical tissue, as well as in blood samples, from the 24 h and 7−day groups. Each hypoxia condition (untreated and treated) is compared to its respective control (anesthesia−only) group. Color-coding reflects the direction and significance of expression changes: shades of green indicate upregulation, shades of red indicate downregulation, and gray indicates non-significant changes (*p* > 0.05). Significance levels are represented according to the adjacent legend. For general analysis, *n* = 5–14, for sex specific subgroups, *n* = 3–7.

In the hippocampus 24 h after hypoxia, CCR7 (Figure 6) is significantly (*p* = 0.0021) upregulated in the treated 24 h treated hypoxia group (4.062 × 10^−5^± 2.887 × 10^−5^). The untreated hypoxia (1.329 × 10^−5^ ± 5.343 × 10^−6^) group shows no differences compared to the control group (8.043 × 10^−6^ ± 4.791 × 10^−6^). In the 7-day group for CCR7, a significant (*p* = 0.0045) increase was seen in the untreated hypoxia group (4.988 × 10^−5^ ± 4.799× 10^−5^). In the other two experimental groups, no significant difference compared to the control could be detected.

*Versican* showed no differences in the untreated hypoxia groups (0.008849 ± 0.003907) but is significantly (*p* < 0.0001) upregulated in the 24 h treated hypoxia group (0.02163 ± 0.01260). This effect is seen in the overall group as well as in the sex-specific subgroups. In the 7-day group, a significant (*p* = 0.0383) increase is observed for *Versican* in the untreated total hypoxemia group (0.02644 ± 0.003936).

The clinical marker S100B showed a significant upregulation (*p* < 0.0001) in the group of 24 h treated hypoxia rats (4.238 ± 2.231) compared to the control rats (0.6386 ± 0.3427). This effect is observed for S100B in both male (*p* < 0.0001) and female (*p* = 0.0073) groups. In the untreated 24 h hypoxia group, there was no change compared to control. In the 7-day group, S100B was significantly (*p* = 0.0243) upregulated in the total treated hypoxia group (8.025 ± 8.044), while the sex-specific subgroups show no changes in regulation compared to control.

#### 2.6.2. Cerebellum

We observed a significant (*p* = 0.0293; *p* = 0.0011) downregulation of *TNF-α* (Figure 6) in both the untreated (4.766 × 10^−5^ ± 2.007 × 10^−5^) and treated hypoxia groups (2.734 × 10^−5^ ± 2.201 × 10^−5^). The sex-specific analysis shows a significant (*p* = 0.0283) downregulation only in the female treated hypoxia group (2.863 × 10^−5^ ± 1.869 × 10^−5^). All other subgroups show no significant difference in the regulation of *TNF-α*.

*Versican* showed a significant (*p* < 0.0001) upregulation in the whole group (0.02694 ± 0.01140) and in the female treated hypoxia group (*p* = 0.0189; 0.02800 ± 0.01237). There are no significant differences between the anesthesia group and the untreated hypoxia group. In the 7-day group, a significant (*p* = 0.0390) downregulation of *Versican* in the treated hypoxia group (0.01847 ± 0.007186) compared to the control group (0.03232 ± 0.01688) was observed.

When analyzing the clinical marker S100B, the only significant changes were observed in the 24 h group. S100B showed a highly significant (*p* < 0.0001) upregulation in the treated hypoxia group (2.114 ± 0.7348) compared to the control group (0.4455 ± 0.1956), whereas the untreated hypoxia group (0.8149 ± 0.4930) was similar to the control group (0.4455 ± 0.1956). This effect was seen in the overall group as well as in both sex-specific subgroups (male: *p* = 0.0012, 2.187 ± 0.8934; female: *p* < 0.0001, 2.041 ± 0.6139).

#### 2.6.3. Cortex

In the 24 h group, no significant regulation of the inflammatory marker *TNF*-α was observed in the untreated (0.00002575 ± 0.00002474) and treated hypoxia (0.00002759 ± 0.00002512) groups compared to the control group (0.00004951 ± 0.00004091). The sex-specific subgroups also showed no significant regulation. In the 7-day group, only *TNF*-α expression in the male treated hypoxia group (0.00001292 ± 0.00000534, *p* = 0.0357) was significantly different to the control group (0.0000475 ± 0.00002174; Figure 6).

There was no significant difference in *Versican* levels between the total untreated (0.00266 ± 0.00388) and treated hypoxia groups (0.001691 ± 0.002119) and the control group (0.007224 ± 0.00496), and in the sex-specific subgroups of the 24 h group. No statistical changes were observed for the marker in the 7-day group, in neither the total group nor the sex-specific subgroups.

The clinical marker S100B showed significant changes in the 24 h group, but no differences were found in the 7-day analysis. There was no significant change in the total untreated hypoxia group (0.848 ± 0.3437), but the female subgroup (1.004 ± 0.3651, *p* = 0.0186) showed significant upregulation and the male subgroup (0.1274 ± 0.1179, *p* = 0.0372) showed significant downregulation. S100B was downregulated in the total (0.3122 ± 0.267, *p* = 0.0325) and male treated hypoxia groups (0.4969 ± 0.249, *p* = 0.0372) compared to the control group (total: 1.129 ± 0.9299; male: 2.02 ± 0.8379), while no significant changes were found in the female subgroup.

### 2.7. Hypoxia Markers in the Blood: 24 h vs the 7-Daygroup After Hypoxemia

In the blood, for the inflammatory marker *TNF-α* (Figure 6), no change was seen in the overall 24 h hypoxia groups (untreated: 0.01446 ± 0.008550; treated: 0.01242 ± 0.001352), but in the female subgroup there was a significant (*p* = 0.0050) increase in expression in the untreated hypoxia group (0.0242 ± 0.009298) compared to the control group (0.01089 ± 0.006127). In the 7-day group after the intervention, *TNF-α* is significantly upregulated in both the untreated (*p* = 0.0205; 0.008366 ± 0.0008872) and treated hypoxia groups (*p* = 0.0236; 0.008251 ± 0.001527). This effect is only observed in the overall groups, but interestingly not in the sex-specific groups.

The marker of extracellular remodeling *Versican* showed a significant (*p* = 0.0004) downregulation in the treated hypoxia group (0.0009421 ± 0.0008398) 24 h after hypoxia. *Versican* is also significantly (*p* = 0.0133; *p* = 0.0189) downregulated in the male-only (0.0008936 ± 0.0005363) and female-only groups (0.0009907 ± 0.001122).

The clinical marker NSE showed only significant changes in blood in the 24 h groups. When analyzing the untreated hypoxia group, a significant increase is observed for the total NSE group (*p* = 0.0415; 4.391 × 10^−5^ ± 5.167 × 10^−5^). In the treated hypoxia group, NSE is highly significantly upregulated in both the total (*p* < 0.0001; 0.0001002 ± 6.941 × 10^−5^) and sex-specific groups (male: *p* = 0.0001; 0.0001395 ± 5.121 × 10^−5^, female: *p* < 0.0001; 4.852 × 10^−5^ ± 1.307 × 10^−5^).

## 3. Discussion

Global cerebral hypoxia triggers a cascade of adaptive and maladaptive responses, including altered neuronal firing patterns, neurotransmitter imbalances, and immune system activation [20,21,22]. While these responses aim to restore homeostasis, they can also exacerbate neuronal damage and functional impairments [23]. Edaravone, a compound with established neuroprotective and antioxidative properties, has shown efficacy in reducing ischemic injury by mitigating neuronal damage and cognitive impairment [24].

### 3.1. Motor Coordination and Activity

Exploratory behavior and tactile curiosity were transiently affected by global cerebral hypoxia, with untreated animals showing an early decline in motor coordination. Edaravone treatment did not result in sustained behavioral enhancement across the general cohort but suggested an acute neuroprotective effect. This is in line with other studies reporting less motor deficits after edaravone treatment in rats and mice undergoing hypoxic–ischemic brain injury [25,26].

Sex-specific differences in response to hypoxia and edaravone treatment were evident, with males showing a 24 h protective effect and females exhibiting greater baseline resistance to hypoxic injury. These findings align with previous studies indicating that male infants are more vulnerable to hypoxic–ischemic injury than females, a pattern supported by rodent models of neonatal hypoxia–ischemia [27]. Explanations for this are diverse and hypothetical; however, gonadal hormones, sex-specific pharmacodynamics, and neuroinflammatory responses may be involved [27,28]. The observed resistance to hypoxia and increased activity by female rats may also reflect an anxiety-related compensatory mechanism [29], potentially mediated by hippocampal dysfunction within the stress-regulating HPA axis [30].

### 3.2. Morphological Integrity

Despite the known susceptibility of high metabolic brain regions like the hippocampus, cerebellum, and primary motor cortex to hypoxic injury, no significant structural degeneration was observed based on neuronal density or cytoarchitecture (Figure A1, Figure A2, Figure A3 and Figure A4). This suggests that functional deficits post-hypoxia may result from subtle or transient cellular dysfunctions rather than overt neuronal loss. The preservation of structural integrity might reflect adaptive neuroprotective mechanisms, such as the upregulation of cerebral blood flow and the release of neuroprotective proteins under mild hypoxic conditions [31,32].

### 3.3. Neuroinflammation and Microglial Activity

Microglial activation, assessed via *Iba1* immunofluorescence, revealed region- and time-dependent alterations following global cerebral hypoxia. A marked reduction in Iba1 signal was observed in the 24 h group, indicating transient suppression of microglial activity. In contrast, a significant rebound activation of microglia was observed in the 7-day untreated hypoxia group, suggesting delayed immune responses that may contribute to neuronal protection and regeneration [20]. Edaravone treatment did not significantly differ from the control group at the early stage. Additionally, the analysis of Tuj1/Iba1 double-positive cells in hippocampal subregions and the M1 region of the cortex did not reveal significant differences, suggesting that the overall microglial response may not be sufficiently captured by these markers alone.

### 3.4. Neuronal Survival and Apoptosis

Co-staining with Tuj1 and activated Caspase-3 revealed no significant increase in apoptotic neurons in the hippocampus or primary motor cortex at 24 h or 7 days post-intervention (Figures S1 and S2). This may reflect the resilience of mature neurons to the global cerebral hypoxia model used or limitations in detecting transient apoptotic phases as observed in other studies [33]. Interestingly, the cerebellum exhibited an increase in double-positive cells in males post-hypoxia, possibly indicating increased apoptosis or altered clearance mechanisms. These sex-specific differences emerged in the 7 days post-intervention, with males in the hypoxia group showing an increase in Casp3+/Tuj1+ neurons, suggesting a delayed apoptotic response or an edaravone-linked pro-apoptotic effect in males [34].

### 3.5. White Matter Integrity and Oligodendrocyte Function

Myelination status, assessed via MBP staining, largely remained stable across chosen regions and time points. However, significant loss of MBP signal in the CA2 subregion of the hippocampus in the 24 h group treated with edaravone suggested early demyelination, indicating a region-specific effect of edaravone on oligodendrocyte function. In the cerebellum, the MBP signal was significantly higher in the untreated hypoxia group than in the edaravone-treated 24 h group, suggesting reactive or compensatory remyelination as an only early effect, given that the differences could not be detected after 7 days. These findings align with previous studies demonstrating the protective effects of edaravone on white matter integrity [35,36]. Additionally, the integrated density of MBP immunoreactivity in the M1 region of the cortex did not show significant differences (Figure S3), suggesting that cortical myelination may be less susceptible to hypoxic damage under the experimental conditions used.

### 3.6. Molecular Mechanisms

qPCR analysis provided insights into the molecular consequences of global cerebral hypoxia, particularly regarding inflammatory responses, extracellular matrix (ECM) remodeling, and the expression of clinical biomarkers. In the hippocampus, inflammation-related genes such as Il1β and TNF-α remained largely unchanged, except for a notable upregulation of CCR7. ECM-related markers like TGF-β and *Versican* were upregulated, particularly in the treated group, indicating early tissue remodeling that may contribute to protective or regenerative processes. Cell culture studies have shown evidence that treatment with edaravone can influence the remodeling process of the ECM, using TGF-β as a marker [37]. Hippocampal expression of MBP and Iba1 in the untreated 24 h and 7-day group also confirmed oligodendrocyte involvement and microglial activation, which is in line with studies demonstrating an increase 24 h after hypoxia, reaching maximum levels after 7 days [38,39]. The clinical markers S100B and NSE were significantly regulated particularly in the treated group, reinforcing their value in detecting acute neuronal injury. Heatmap visualizations of gene expression changes in the hippocampus, cerebellum, cortex, and peripheral blood (Figures S4–S7) revealed region- and time-specific patterns of upregulation and downregulation, highlighting the complex interplay of molecular responses to hypoxia and edaravone treatment.

In the cerebellum, both Il1β and TNF-α were significantly downregulated in treated 24 h animals, implying a more rapid resolution or suppression of inflammation compared to the hippocampus. Corresponding results have been observed in rats after traumatic brain injury following edaravone treatment [40]. *Versican* showed the same trend as in the hippocampus, suggesting a contribution to protective or regenerative processes. Significant higher expression of MBP was shown under edaravone treatment, suggesting stronger and faster remyelination and maturation of oligodendrocytes associated with treatment [41]. In addition, the significantly greater increase in male animals suggests a sex-specific activation of oligodendrocytes.

### 3.7. Limitations and Future Directions

Summarized, edaravone exhibits transient neuroprotective effects on motor behavior and early immune responses, particularly in the cerebellum and the hippocampus. As morphological damage was mild after hypoxia, edaravone did not result in neuronal protection; however, functional impairments occurred despite preserved cytoarchitecture, suggesting subtle or transient disruptions. Microglial activity was suppressed early in the treated groups and activated later in the untreated hypoxic brains. Gene expression analysis revealed region-specific, time-dependent, and sex-specific changes, including the early upregulation of markers such as CCR7, S100B, and NSE in treated animals. This highlights the effect of edaravone on ECM remodeling, inflammation, and astrocytic activation, while GAPDH may be regulated under hypoxia. While some studies suggest GAPDH may potentially be upregulated under hypoxic conditions in some tissues, others confirm its stability. Future studies should employ alternative reference genes or multi-gene normalization strategies to ensure accurate quantification of gene expression changes.

The variation in the efficacy of edaravone between different brain regions suggests that tissue-specific responses may affect translational outcomes. Sex-specific responses highlight underlying biological differences, warranting further analysis of hormonal, neurochemical, and inflammatory profiles. Seven-day recovery, degeneration patterns, and chronic conditions require further investigation. Future studies should also focus on mitochondrial function, blood–brain barrier integrity, and structural changes during edaravone treatment using techniques such as electron microscopy. Molecular analyses of neuroinflammatory and oxidative stress markers would further validate the therapeutic potential of edaravone.

## 4. Materials and Methods

### 4.1. Animals

All procedures were carried out under established standards of the German federal state of North Rhine-Westphalia, in accordance with the European Communities Council Directive 2010/63/EU on the protection and care of animals used for scientific purposes. Permission to conduct these experiments was granted by the North Rhine-Westphalia State Office for Consumer Protection and Food (LAVE-NRW), file no. 81-02.04.2021.A452.

The Wistar rats used in this study were bred in-house at the animal facility of medicine of Ruhr University Bochum, following the selection of breeding pairs based on age, weight, and overall health. Adhering to the principles of the 3Rs, the breeding program aimed to minimize unnecessary animal use by breeding only the required number of animals for the study, resulting in a lack of influence over sex distribution. For female rats it was ensured that they were not pregnant before or during the experiments. Animal care procedures followed ethical guidelines and regulations. Rats were kept under a 12 h light/dark cycle and had ad libitum access to food and water.

### 4.2. Experimental Setup—Induction of Global Cerebral Hypoxia

The experimental setup was performed as described by Stahlke et al. (2024) [19]. Summarized, animals were anesthetized with ketamine and midazolam i.p. before they obtained rocuronium i.v., leading to severe respiratory insufficiency. An oxygen saturation of 60% was targeted and measured via pulse oximeter and was aimed to be maintained for 12 min. In general, “hypoxia” was defined as 10% reduction from baseline blood oxygen saturation (SpO_2_), ensuring all animals reached cerebral hypoxia. While we aimed for continuous 60% SpO_2_, physiological variability among animals resulted in fluctuations around this target but remained in all animals below −10% from baseline for 12 min and never fell below 50% to avoid lethal cardiac complications. Then, sugammadex i.v. was administered to stop the effect of rocuronium immediately. In addition to the two control groups, a third group underwent treatment with edaravone. Rats in the experimental group received a 750 mg/kg intraperitoneal injection of edaravone directly after the “hypoxic interval”. In total, four groups were examined: (1) control—no hypoxia + no edaravone treatment, (2)—no hypoxia + edaravone treatment, (3) untreated hypoxia—12 min hypoxia + no edaravone treatment, and (4) treated hypoxia—12 min hypoxia + edaravone treatment.

The animals were either terminated 24 h or 7 days after induction of anesthesia in order to analyze the neuroprotective effects at two time points. Rats subjected to the 7-day experimental schedule were treated each day by i.p. injection of edaravone at the same time. After termination, blood and brain tissues were collected for further immunohistochemical and molecular analysis.

### 4.3. Behavior Tests

The exploratory behavior and motor coordination of the rats was assessed in the 7-day group. The rats were therefore placed in an upright plastic cylinder for 3 min. A lid prevents the rat from jumping out of the cylinder. During this time period, the number of forefoot touches was counted, as well as the time the rat stood with their paws above shoulder height. The behavior tests were recorded by a camera for analysis. One day before induction of hypoxia (p − 1), the first behavior test was performed and used as the baseline. During the experimental period, further video recordings were made one (p + 1), three (p + 3) and six (p + 6) days after the induction of hypoxia or the start of the experimental setup.

### 4.4. RNA and cDNA Extraction

Blood was extracted with an intracardiac injection, collected, and cDNA was purified from 200 µL of blood by using a NucleoSpin Blood Kit (740951.10, Macherey-Nagel, Düren, Germany) according to the manufacturer’s instructions.

The hippocampus and the cerebellum were dissected from one hemisphere of each brain. The right hemisphere of every animal was used for RNA isolation, of which the whole hippocampus and cerebellum were dissected on ice. Both structures were homogenized completely. The RNA of each area was isolated separately using the NucleoSpin RNA Kit (740,955.50, Macherey-Nagel, Düren, Germany). In deviation from the manufacturer’s instructions, RNA was eluted in only 30 µL of nuclease-free water as a last step. An amount of 1 μg of the resulting RNA was transcribed into cDNA using GoScriptTM Reverse Transcription Mix, Oligo(dT) (A2790—Promega, Madison, WI, USA) according to the manufacturer’s instructions.

### 4.5. Quantitative Real-Time PCR (qRT-PCR)

GoTaq^®^ qPCR Master Mix (A6001, Promega, Madison, WI, USA) was used to perform qRT-PCR according to the manufacturer’s instructions. Blood-derived cDNA was diluted 1:15 and tissue-derived cDNA was diluted 1:20 in nuclease-free water. Expression levels for the genes of interest and for housekeeping gene GAPDH were measured in duplicates. Used primers are listed in Table 1. Quantitative rt-PCRs were performed on a CFX Connect Real Time PCR Detection System (Bio-Rad, Hercules, CA, USA). The X0 method was applied, using the mean ct-value normalized to the housekeeping gene GAPDH, before the results were plotted logarithmically to base 10 using mean and standard deviation (SD).

### 4.6. Cryosectioning

The left hemisphere of every animal was fixed in 4% paraformaldehyde (PFA) (28794.295, VWR Chemicals, Darmstadt, Germany) immediately after preparation. After 24 h the tissue was transferred to 30% sucrose (D(+)-Sucrose pure, A1125, AppliChem, Darmstadt, Germany) and stored at 4 °C.

The complete hemispheres were cut into 14 µm sagittal sections, with three sections per slide grouped in slides of 10. For RNase-free conditions, removable parts of the cryostat (CryoStar NX50, Thermo Scientific, Waltham, MA, USA) were cleaned with NaOH-EDTA. Cryosectioning was performed at a chamber temperature of −20 °C and a stage temperature of −15 °C to −12 °C. In the chamber, the frozen hemispheres were stabilized on the microscope slide with tissue freezing medium (No. 14020108926, Leica Biosystems, Wetzlar, Deutschland). After a 20–30 min acclimatization period, the specimen was placed on the cryostat stage with the upper side facing up and cut. To ensure uniformity between the rats, serial brain slices were created, each labeled with chronological numbers. A total of 40 slides, each containing three brain slices from the individual rat, were created. In total, 120 brain slices per rat were taken, giving an overview over the whole hemisphere.

The serial cryosections for immunohistochemistry were mounted on Superfrost-Plus Adhesion Slides (J1800AMNZ, Thermo Scientific, Waltham, MA, USA) and stored at −20 °C for further use.

### 4.7. Cresyl Violet Staining

For histochemical staining, the cryosections were immersed in cresyl violet staining (7651.1 (Roth, Karlsruhe, Germany)) for 2 min. The slides were then rinsed with tap water to remove any excess stain in two different cuvettes. To destain the slides, they were washed twice with 95% EtOH for 15 s. For further differentiation, the samples were rewashed twice with 100% EtOH for 2.5 min. Then, the slides were placed in a cuvette with 100% xylol (AnalaR NORMAPUR, 28975.325, Sigma-Aldrich, Darmstadt, Germany) for 2.5 min. After differentiation, the stained samples were coverslipped with mounting medium (Eukitt^®^ quick-hardening mounting medium, Sigma-Aldrich, Darmstadt, Germany).

Determining the cell numbers or density in the hippocampus, cerebellum and primary motor cortex.

The CA1 and the CA2 region in cresyl violet-stained slides were analyzed using the same exposure time and objective lens magnification for every slide (BZ-X800, Keyence, Frankfurt, Germany). The obtained pictures were imported to ImageJ Software 1.53 (Schneider, C.A., Rasband, W.S., Eliceiri, K.W. “NIH Image to ImageJ: 25 years of image analysis”. Nature Methods 9, 671-675, 2012). The threshold was adjusted to highlight all nuclei in both CA1 and CA2 regions. A square was drawn around the regions and the mean gray value (representing cell numbers) within this area was calculated. Images for Purkinje cell (PC) counts were acquired in ImageJ 1.53 in a similar way to the procedure described above. For cell counting, the multi-point tool was used to capture every Purkinje cell along the primary fissure. To ensure unbiased assessment, a single observer performed both observations, intentionally blinded to the experimental conditions. For further classification, PCs were divided into three categories according to their cytological characteristics as described in Stahlke et al. (2024) [19]. Again, PCs were counted along the primary fissure of the cerebellum (assisted by the ImageJ multi-point tool and marked by mouse click) and assigned to PC groups I-III. The area of the Purkinje cell body was also outlined using the freehand tool in ImageJ 1.53. The measure function was used to analyze the area, shape descriptors, perimeter, and Feret’s diameter of the cells.

The images of the cells in the primary motor cortex were captured using ImageJ 1.53 software, following a procedure similar to the one described above. To determine the area of the primary motor cortex, a rectangular analysis area was manually defined. This area extended ventrally from the indentation of the cerebral cortex dorsal to the lateral ventricle to the corpus callosum. From the corpus callosum, the area extended in a posterior–inferior direction to the CA2 region of the hippocampus. The area was then delineated dorsally from the cerebral cortex. Within this defined area, the average gray value was calculated, serving as an indirect measure of cell density.

### 4.8. Immunohistochemical Staining

Three different immunohistochemical stainings were performed, using antibodies against Iba1 (microglia), MBP (myelin), and a double staining for Tuj1 (neurons) and Caspase-3 (cell death). Primary antibodies against Iba1 (#019-19741, FUJFILM Wako Pure Chemical Corporation, Japan; 1:1000 in 10% normal goat serum (NGS; G9023, Sigma-Aldrich, Darmstadt, Germany)) and against Cas-3 (D175, RB cell signaling 9661S, Germany; 1:200 in 10% NGS) were obtained from rabbits (rb). In contrast, the antibodies against MBP (A-3 sc376995, Santa Cruz, Heidelberg, Germany; 1:500 in 10% NGS) and Tuj1 (MAB 1195, RD Systems, Wiesbaden, Germany; 1:500 in 10% NGS) were obtained from mice (ms). Corresponding species-specific secondary antibodies were used. The same protocol was used for all three staining procedures. Iba1 and MBP were single stainings (two-day protocol), while Tuj1/Cas-3 required a three-day double-staining protocol.

Tissue sections were outlined with a PapPen (Kisker Biotech GmbH, Steinfurt, Germany) and washed three times with 1 × PBS. All washes were performed at room temperature unless otherwise stated. Permeabilization was performed using 1% Triton X-100 (Roth 3051.3, Karlsruhe, Germany) for 15 min at room temperature, followed by additional PBS washes. Sections were then blocked with 10% NGS for 45 min. After a PBS rinse, primary antibodies were applied and incubated overnight at 4 °C.

For double staining (Tuj1/Cas3), a three-day protocol was used. On day two, sections were washed and incubated for 2 h with the first secondary antibody (goat anti-mouse AF488; A28175, Invitrogen, Fisher, Schwerte, Germany), followed by application of the second primary antibody (anti-Cas3, rabbit) and overnight incubation at 4 °C. On day three, sections were washed and incubated with the second secondary antibody (goat anti-rabbit Cy3) (RF) for 2 h. For single stainings (Iba1 or MBP), only one primary/secondary pair was used, and the protocol was completed in two days.

After secondary antibody incubation, all sections were stained with DAPI (Sigma B1155; 30 min), washed, mounted in Fluoromount, and coverslipped.

Immunostained sections were imaged using a fluorescence microscope (BZ-X800, Keyence, Frankfurt, Germany) at 20× magnification. DAPI (CH1), GFP (CH2: Tuj1, Iba1), and TRITC (CH3: MBP, Cas3) channels were used. A fixed 1 s exposure was applied for MBP; other exposure times were adjusted individually for optimal signal. Iba1- and Tuj1/Cas3-positive cells were quantified using ImageJ.

### 4.9. Statistical Analysis

Data from the study were analyzed using appropriate statistical methods, such as t-tests for differences between groups or analysis of variance (ANOVA) when more than two groups were examined. Prior to statistical analysis, we analyzed whether the data had a Gaussian distribution, which was the case. Results are reported as mean ± standard deviation (SD) and significance levels are set at *p* < 0.05 indicated by *, *p* < 0.001 indicated by **, or *p* < 0.0001 indicated by ***. Statistical outliers were removed via ROUT method with Q-value set to 5% if necessary. GraphPad Prism9 (GraphPad Software, San Diego, California United States) was used to plot the data.

## 5. Conclusions

This study used the innovative rat hypoxia model to evaluate neuroprotective effects of edaravone under various conditions, such as sex differences, treatment durations, and behavioral and blood-based outcomes. Our findings highlight the potential for personalized stroke therapy by considering individual variability in treatment responses.

Edaravone exhibits transient neuroprotective effects on motor behavior and early immune responses, particularly in the cortex and hippocampus. Histological analyses revealed no gross morphological damage following hypoxia, and edaravone did not result in measurable structural protection at the neuronal level. However, functional impairments occurred despite preserved cytoarchitecture, suggesting subtle or transient disruptions. Sustained functional improvements were limited and region-specific. Microglial activity was suppressed early in the treated groups and activated later in the untreated hypoxic brains, indicating that edaravone modulates immune responses to stabilize the neuroinflammatory environment rather than suppressing it outright.

Gene expression analysis revealed region-specific, time-dependent, and sex-specific changes, including the early upregulation of markers such as CCR7, S100B, and NSE, in treated animals. This highlights edaravone’s modulation of ECM remodeling, inflammation, and astrocytic activation.

Notably, males and females often exhibited contrasting responses following hypoxia. In the behavior tests, males were more susceptible to hypoxic damage and did not show full recovery despite early treatment with edaravone, while females showed higher baseline resistance and better functional recovery. In the 7-day group, edaravone led to an increase in apoptotic markers in the hippocampus of female animals, indicating sex-specific differences in cell death mechanisms and the immune response.

Taking sex differences into account in both research and clinical practice is essential to ensure effective, personalized treatment strategies for stroke patients. Future studies should focus on elucidating the underlying mechanisms of these sex-specific responses and exploring long-term outcomes to further validate the therapeutic potential of edaravone.

## Figures and Tables

**Table 1:** List of primers used to validate successful global cerebral hypoxia.

Primer	Forward	Reverse
Il1β	GACAAGAGCTTCAGGAAGGCA	CCACGGGCAAGACATAGGTAG
TNF-α	GCTCCCTCTCATCAGTTCCA	GCTACGGGCTTGTCACTC
CCR7	TGGTCATTTTCCAGGTGTGCT	TACAGGGTGTAGTCCACGGT
*Versican*	CGCCTAAGACACTACGTATGCTTGT	TTGGTCCTATGTTGACTGTTTCTCA
TGF-β	CTGCTGACCCCCACTGATAC	AGCCCTGTATTCCGTCTCCT
Iba1	TGC AGC CTC ATC GTC ATC TC	TTT TCC TCC CTG CAA ATC CTT
MBP	GGC AAG GAC TCA CAC ACA AGA A	CTT GGG TCC TCT GCG ACT TC
NSE	GGGGCACTCTACCAGGACTTTG	GTTCCGGTGTTCAGGCAAGCAG
S100B	CTGGAGAAGGCCATGGTTGC	CTCCAGGAAGTGAGAGCT
GAPDH	GGGTGTGAACCACGAGAAAT	ACTGTGGTCATGAGCCCTTC

## Data Availability

The raw data supporting the conclusions of this article will be made available by the authors on request.

## Supplementary Materials

The following supporting information can be downloaded at: https://www.mdpi.com/article/10.3390/ijms26189019/s1.

## Abbreviations

The following abbreviations are used in this manuscript:

3Rs	Replacement, Reduction, and Refinement
AIF	Apoptosis-Inducing Factor
ALS	Amyotrophic Lateral Sclerosis
BZ	Betz Cell
CA1/CA2	Cornu Ammonis Region 1/2 (Hippocampal Subfields)
Casp3	Caspase-3 (Cleaved/Activated Form)
CCR7	C-C Chemokine Receptor Type 7
Ct	Cycle Threshold (in qPCR)
CV	Cresyl Violet
ECM	Extracellular Matrix
EPO	Erythropoietin
GAPDH	Glyceraldehyde-3-Phosphate Dehydrogenase (Housekeeping Gene)
GFP	Green Fluorescent Protein
HIF/HIF-1α/HIF-2α	Hypoxia-Inducible Factor 1 and 2 Alpha
HPA	Hypothalamic–pituitary–adrenal axis
Iba1	Ionized Calcium-Binding Adapter Molecule 1
iNOS	Inducible Nitric Oxide Synthase
IL-1β/Il1β	Interleukin-1 Beta
JNK	c-Jun N-Terminal Kinase
LAVE-NRW	Landesamt für Verbraucherschutz und Ernährung Nordrhein-Westfahlen
MBP	Myelin Basic Protein
MD2	Myeloid Differentiation Protein 2
M1	Primary Motor Cortex
ML	Molecular Layer
NGF	Nerve Growth Factor
NGFI-A	Nerve Growth Factor-Induced Protein A
NSE	Neuron-Specific Enolase
NSS	Neurological Severity Score
NF-κB	Nuclear Factor Kappa-Light-Chain-Enhancer of Activated B Cells
OPCs	Oligodendrocyte Precursor Cells
OGD/UGD	Oxygen–Glucose Deprivation
P1/P3/P6	Postoperative Day 1/3/6
PARP-1	Poly (ADP-Ribose) Polymerase 1
PC	Purkinje Cell
PCL	Purkinje Cell Layer
PI3K	Phosphoinositide 3-Kinase
PRF/mm	Purkinje Cells per Millimeter
rb	Rabbit (Source of Antibody)
ROUT	Robust Regression and Outlier Removal
ROS	Reactive Oxygen Species
RNS	Reactive Nitrogen Species
rt-PA	Recombinant Tissue Plasminogen Activator
S100B	S100 Calcium Binding Protein B
TBI	Traumatic Brain Injury
TGF-β/TGFβ	Transforming Growth Factor Beta
TNF-α/TNFα	Tumor Necrosis Factor Alpha
TRITC	Tetramethylrhodamine
Tuj1	Neuronal Class III β-Tubulin

## Appendix A

### Appendix A.1. Cresyl Violet-Based Assessment of Neuronal Structure in the Hippocampus

In the 24 h group, no significant differences in mean gray values were observed between the control (220.1 ± 16.04/215.8 ± 13.77), untreated hypoxia (197.3 ± 13.48/184.9 ± 14.07), and edaravone-treated hypoxia groups (192.3 ± 26.84/183.5 ± 31.29) in either CA1 or CA2. Likewise, in the 7-day group, the mean gray value analysis revealed no significant differences across the subgroups (CA1 control: 211.0 ± 1.94; untreated: 197.5 ± 19.15; treated: 206.3 ± 13.32/CA2 control: 195.9 ± 17.78; untreated: 188.0 ± 22.85; treated: 195.0 ± 14.95).

### Appendix A.2. Morphological Alterations of Neuronal Integrity in the Cerebellum

Quantitative analysis of Purkinje cell counts in the primary fissure showed no significant differences between subgroups in either the 24 h (control: 14.14 ± 4.13 cells; untreated: 14.27 ± 3.41; treated: 17.73 ± 2.36) or the 7-day group (control: 20.12 ± 1.99; untreated: 20.93 ± 9.36; treated: 18.52 ± 3.32).

Secondly, the length of the primary fissure as a structural parameter of cerebellar morphology after global cerebral hypoxia was analyzed. In the 24 h group, no significant differences in fissure length were observed between subgroups (control: 6228 ± 1033 µm; untreated: 6167 ± 1931 µm; treated: 5063 ± 1162 µm). Similarly, the 7-day post-intervention group showed no detectable changes in primary fissure length across all experimental conditions (control: 4778 ± 769.1 µm; untreated: 5006 ± 1177 µm; treated: 4724 ± 499.1 µm).

Purkinje cell density was quantified as the number of Purkinje cells per micrometer of the Purkinje layer (PRF/mm). No significant difference in PRF/mm was observed in both hypoxia groups compared to controls in the 24 h intervention (control: 14.14 ± 4.13 PRF/mm; untreated: 14.27 ± 3.41; treated: 17.73 ± 2.36). Similarly, for the 7-day animals, PRF/mm values remained comparable across all experimental groups (control: 20.12 ± 1.99 mm; untreated: 20.93 ± 9.36; treated: 18.52 ± 3.32).

As mentioned in the method section, the Purkinje cells were divided into three subtypes, which represent morphologically intact cells (type I), possibly damaged cells characterized by enlarged soma and intracellular vacuolization (type II), and ruptured, necrotic cells (type III).

In the quantitative analysis of the 24 h group, no significant difference in the amount of type I cells was observed (control: 49.57 ± 13.20 cells; untreated: 45.11 ± 21.50; treated: 51.83 ± 12.50). In the quantitative analysis of the type II Purkinje cells, no significant difference was detected between the subgroups (control: 22.29 ± 6.40 cells; untreated: 23.44 ± 8.71; treated: 24.58 ± 8.98), either. Similarly, the quantitative analysis of the type III cells did not show any significant changes in the subgroups as well (control: 14.00 ± 11.50 cells; untreated: 17.67 ± 10.26; treated: 12.50 ± 4.25).

In the quantitative analysis of the 7-day group, no significant difference in the amount of type I cells was observed (control: 47.20 ± 14.04 cells; untreated: 59.89 ± 19.30; treated: 45.63 ± 12.94). Similarly, no significant differences were detected in the number of type II cells between groups (control: 29.20 ± 8.50 cells; untreated: 24.33 ± 10.87; treated: 26.25 ± 10.53). Analysis of type III cells also showed no significant variation across the subgroups (control: 18.80 ± 5.93 cells; untreated: 14.22 ± 6.82; treated: 15.63 ± 6.09).

### Appendix A.3. Cresyl Violet-Based Analysis of the Primary Motor Cortex

The quantitative analysis of the M1 region in the 24 h group showed no significant difference in the subgroups (control: 25.75 ± 5.56; untreated: 36.29 ± 9.01; treated: 29.75 ± 13.71).

Likewise, in the quantitative analysis of the mean gray value in the 7-day group, we observed no significant changes between the subgroups (control: 29.00 ± 15.56; untreated: 27.33 ± 15.81; treated: 23.38 ± 12.84).

In addition, the size in µm^2^ of the M1 region was measured and compared between the subgroups. In neither the 24 h group (control: 2.31 × 10^6^ ± 608830; untreated: 2.14 × 10^6^ ± 464204; treated: 183 × 10^6^ ± 439467) nor the 7-day group (control: 2.12 × 10^6^ ± 305779; untreated: 1.78 × 10^6^ ± 229831; treated: 1.82 × 10^6^ ± 259088) were any significant differences detected.
