# Supplementary material for: Therapeutic Potential of Edaravone for Neuroprotection Following Global Cerebral Hypoxia
Source: Int J Mol Sci. 2025 Sep 16;26(18):9019. doi: 10.3390/ijms26189019 (PMC12469665; doi:10.3390/ijms26189019)
Supplement: Supplementary file 1 [file ijms-26-09019-s001.zip › ijms-3822299 -Supplement.pdf]

International Journal of
Molecular Sciences

MDPI

Supplement

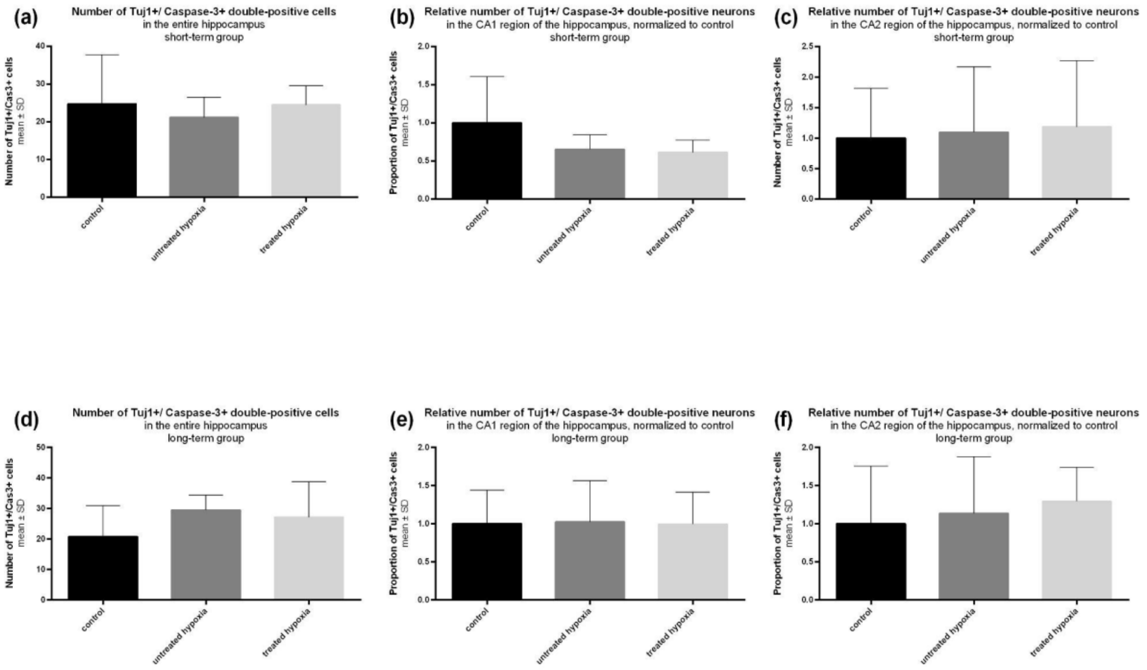

Figure S1. Relative number of TUJ1/caspase 3 double-positive cells in hippocampal subregions.

Bar graphs show the proportion of TUJ1/caspase 3 double-positive cells normalized to the control group in short-term (a–c) and long-term (d–f) animals.

(a, d) entire hippocampus, (b, e) CA1 region, (c, f) CA2 region

Each graph compares three experimental conditions: control, untreated hypoxia, and treated hypoxia. Data are presented as mean ± SD.

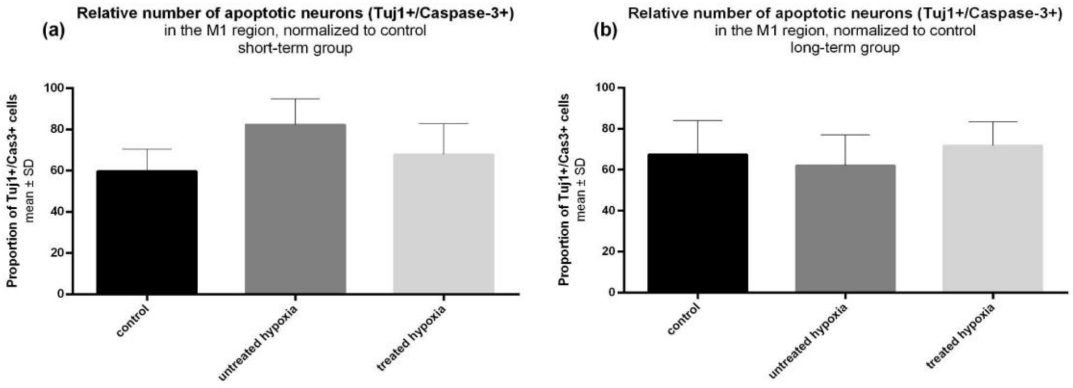

Figure S2. Relative number of TUJ1/caspase 3 double-positive cells in the M1 region of the cortex.

Bar graphs show the proportion of TUJ1/caspase 3 double-positive cells normalized to the control group in (a) the short-term group and (b) the long-term group. Each graph compares three experimental conditions: control, untreated hypoxia and treated hypoxia. Data are presented as mean ± SD.

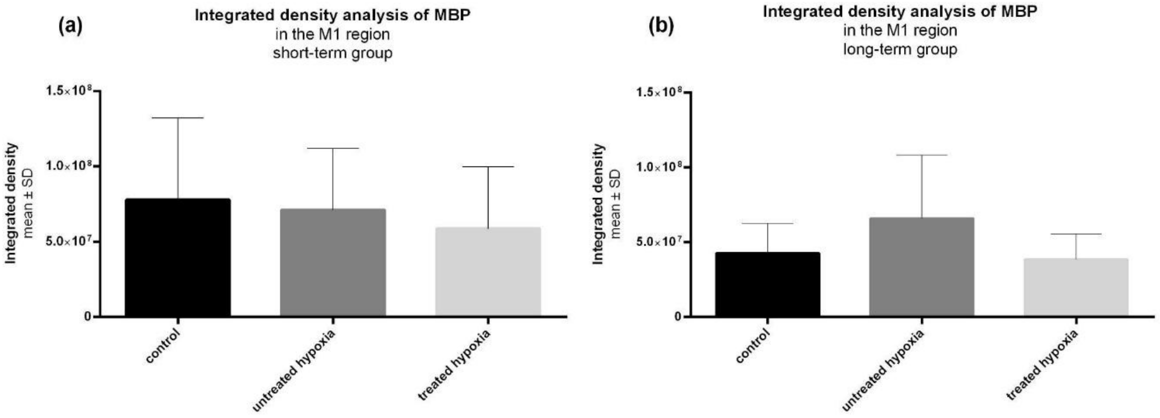

Figure S3. Integrated density of MBP immunoreactivity in the M1 region of the cortex.
Bar graphs show the integrated density of MBP staining in the M1 region for (a) the short-term group and (b) the long-term group. Each graph compares three experimental conditions: control, untreated hypoxia and treated hypoxia. Data are presented as mean ± SD.

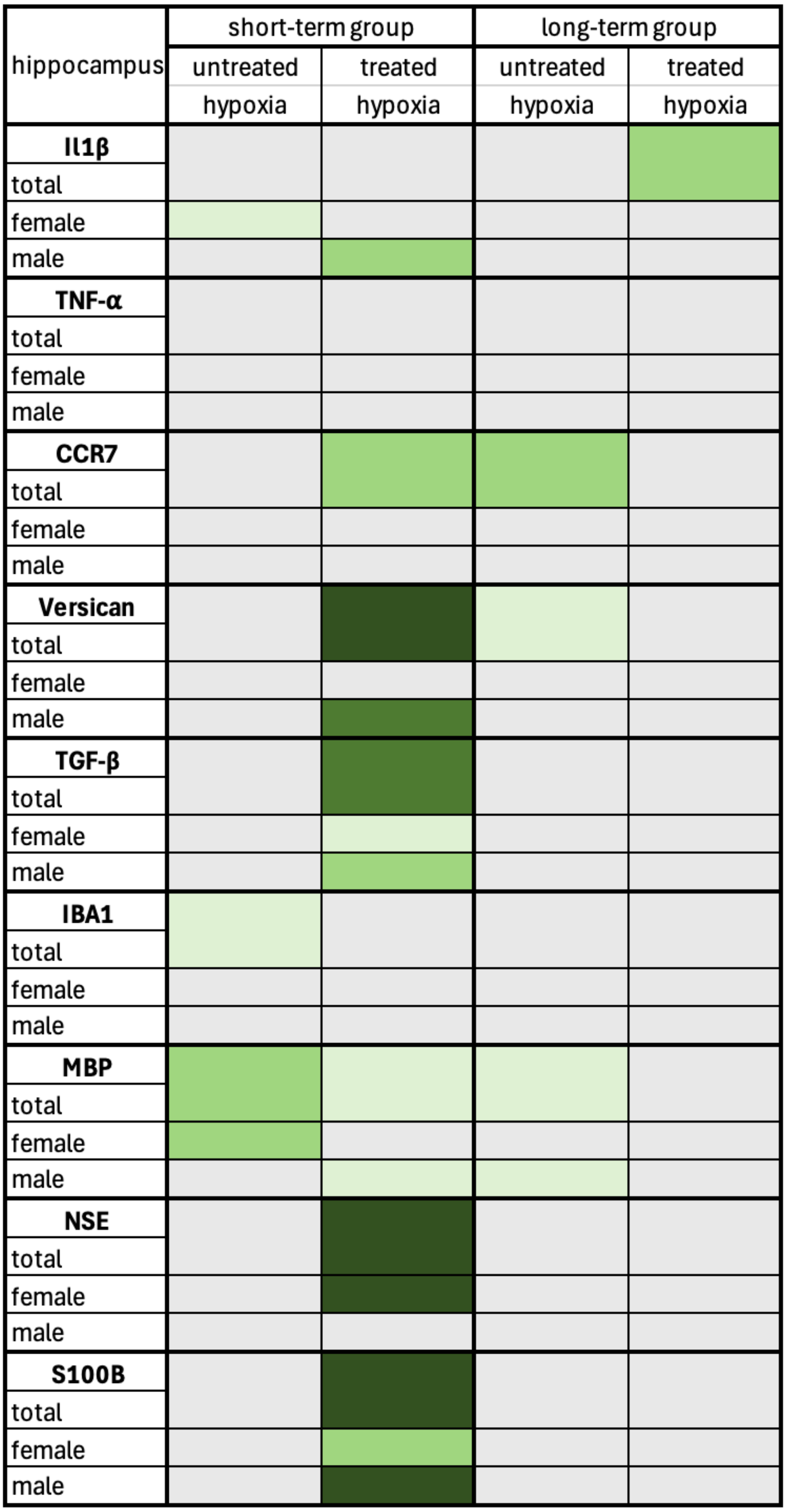

Figure S4. Heatmap visualization of gene expression changes in the hippocampus following hypoxia.

Heatmap showing relative mRNA expression levels of screened hypoxia markers in hippocampal tissue from the short-term and long-term groups. Each hypoxia condition (untreated and treated) is compared to its respective control (anesthesia-only) group. Color coding reflects the direction and significance of expression changes: shades of green indicate upregulation, shades of red indicate downregulation, and grey indicates non-significant changes ($p > 0.05$).

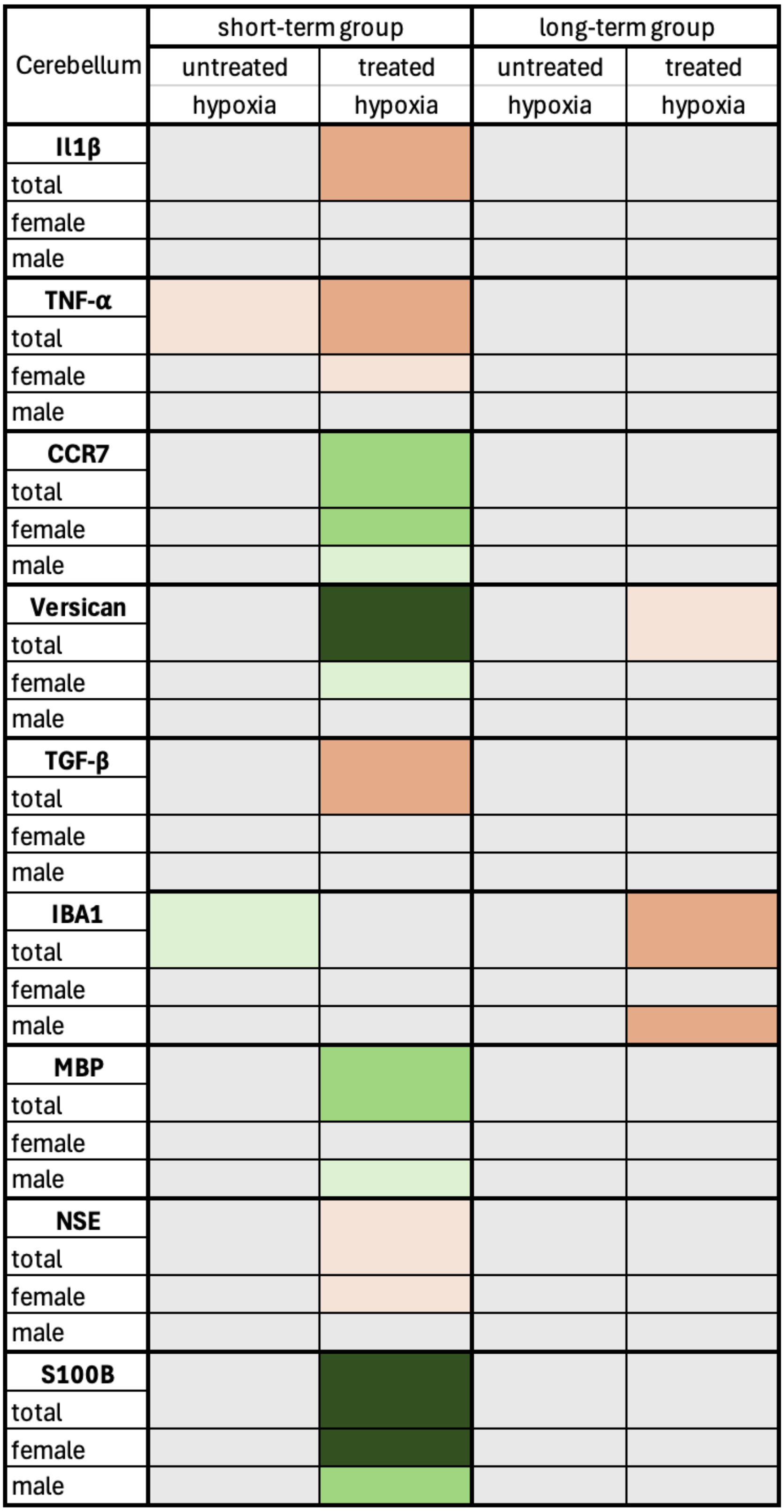

Figure S5. Heatmap visualization of gene expression changes in the cerebellum following hypoxia.

Heatmap showing relative mRNA expression levels screened hypoxia markers in cerebellar tissue from the short-term and long-term groups. Each hypoxia condition (untreated and treated) is compared to its respective control (anesthesia-only) group. Color coding reflects the direction and significance of expression changes: shades of green indicate upregulation, shades of red indicate downregulation, and grey indicates non-significant changes ($p > 0.05$).

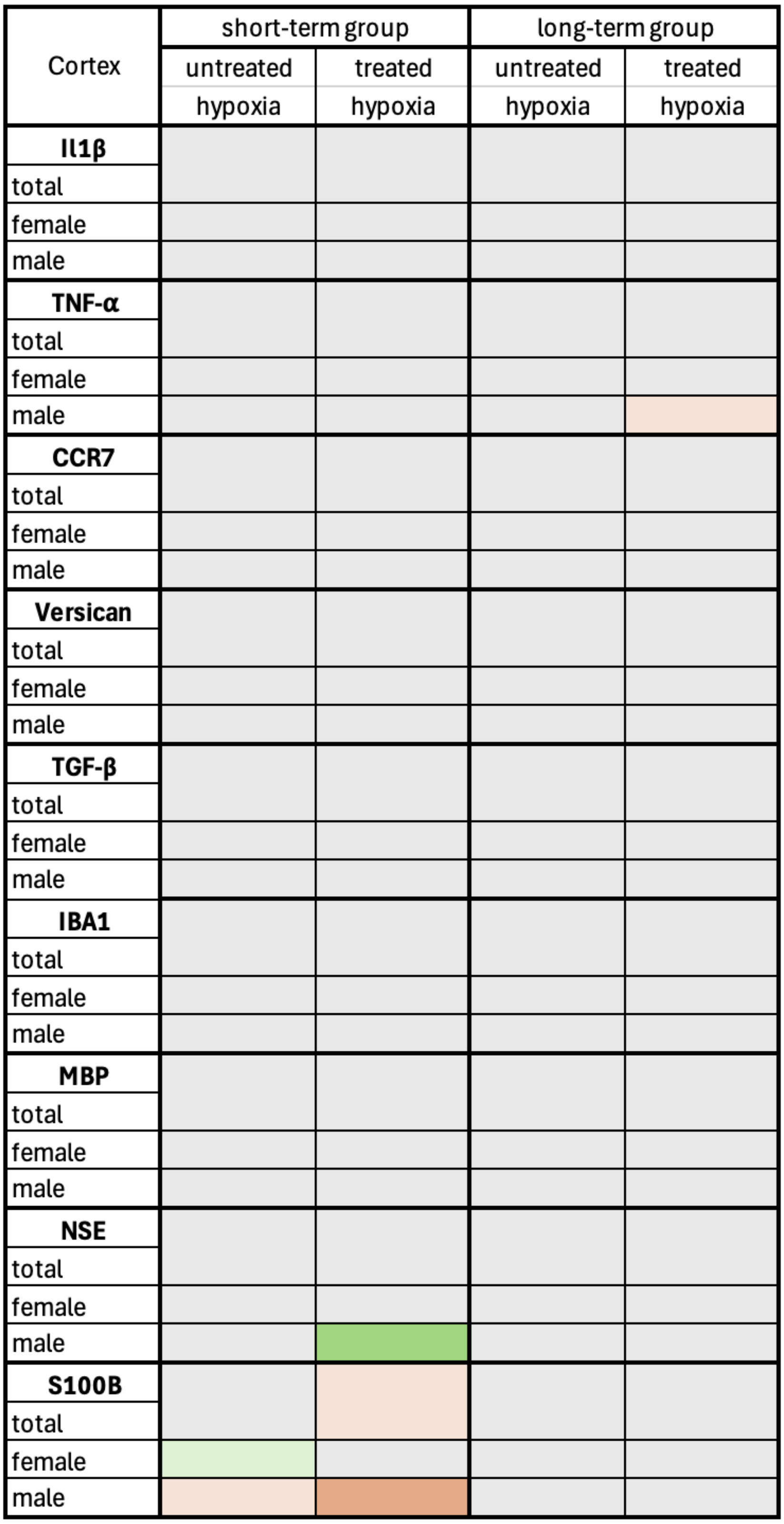

Figure S6. Heatmap visualization of gene expression changes in the cortex following hypoxia.

Heatmap showing relative mRNA expression levels screened hypoxia markers in cortex tissue from the short-term and long-term groups. Each hypoxia condition (untreated and treated) is compared to its respective control (anesthesia-only) group. Color coding reflects the direction and significance of expression changes: shades of green indicate upregulation, shades of red indicate downregulation, and grey indicates non-significant changes ($p > 0.05$).

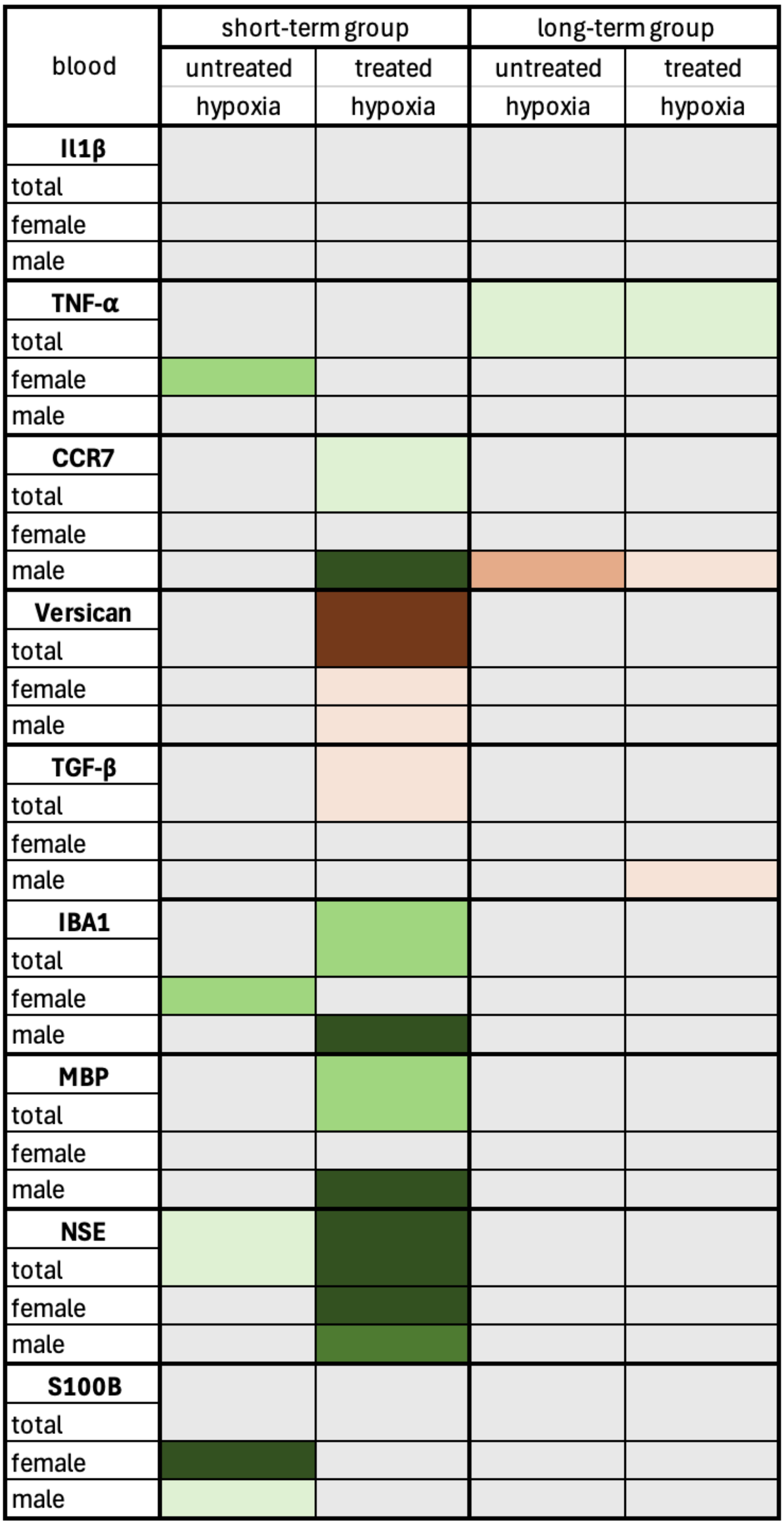

Figure S7. Heatmap visualization of gene expression changes in peripheral blood following hypoxia.

Heatmap showing relative mRNA expression levels of screened hypoxia markers from the short-term and long-term groups. Each hypoxia condition (untreated and treated) is compared to its respective control (anesthesia-only) group. Color coding reflects the direction and significance of expression changes: shades of green indicate upregulation, shades of red indicate downregulation, and grey indicates non-significant changes ($p > 0.05$).
